# PrognoScan: a new database for meta-analysis of the prognostic value of genes

**DOI:** 10.1186/1755-8794-2-18

**Published:** 2009-04-24

**Authors:** Hideaki Mizuno, Kunio Kitada, Kenta Nakai, Akinori Sarai

**Affiliations:** 1 Pharmaceutical Technology Department, Kamakura Research Laboratories, Chugai Pharmaceutical Co Ltd, Kamakura, Kanagawa, Japan; 2 Department of Biosciences and Bioinformatics, Kyushu Institute of Technology, Iizuka, Fukuoka, Japan; 3 Human Genome Center, Institute of Medical Science, University of Tokyo, Minato-ku, Tokyo, Japan

## Abstract

**Background:**

In cancer research, the association between a gene and clinical outcome suggests the underlying etiology of the disease and consequently can motivate further studies. The recent availability of published cancer microarray datasets with clinical annotation provides the opportunity for linking gene expression to prognosis. However, the data are not easy to access and analyze without an effective analysis platform.

**Description:**

To take advantage of public resources in full, a database named "PrognoScan" has been developed. This is 1) a large collection of publicly available cancer microarray datasets with clinical annotation, as well as 2) a tool for assessing the biological relationship between gene expression and prognosis. PrognoScan employs the minimum *P*-value approach for grouping patients for survival analysis that finds the optimal cutpoint in continuous gene expression measurement without prior biological knowledge or assumption and, as a result, enables systematic meta-analysis of multiple datasets.

**Conclusion:**

PrognoScan provides a powerful platform for evaluating potential tumor markers and therapeutic targets and would accelerate cancer research. The database is publicly accessible at .

## Background

A number of genes are recognized as being potentially relevant to cancers. One way to evaluate such genes is to assess their relationship to prognosis. At present, many cancer microarray datasets with clinical annotation have become available in the public domain and provide vast opportunities to link gene expression to prognosis. However, the data are not easy to access and analyze without an effective analysis platform.

Standard survival analysis consists of two steps: 1) grouping patients and 2) comparing the risk difference of the groups. When conducting survival analysis based on continuous measurement such as gene expression, determination of the appropriate cutpoints for groupings remains a critical and difficult task. Thus, although two pioneer databases, ITTACA [1] and REMBRANDT , have provided survival analysis functionality with user defined cutpoints for several focused cancer microarray datasets, researchers without prior biological knowledge or assumptions for the gene may end up using an arbitrary threshold (e.g. median, tertile, quartile) that does not necessarily reflect the biology of the gene or may laboriously test a number of possible cutpoints.

The minimum *P*-value approach is a comprehensive method to find the optimal risk separation cutpoint in continuous measurements and have shown the utility in the analyses of tumor size [2], cell cycle phase estimation measurement [3], and gene copy number [4]. In addition, it is intuitive for oncologists, and thus, a systematic application of this approach to gene expression from microarray seems logical. Recent studies have reported expression thresholds at which the gene becomes a contributor to the development of the cancer such as Bub1 for tumorigenesis [5], HOXB4 for cellular transformation [6], and MYC for tumor maintenance [7], and provided a rationale for the application to gene expression. Thus, we developed "PrognoScan", a database featuring a large collection of publicly available cancer microarray datasets with clinical annotation and a tool for assessing the relationship between gene expression and prognosis using the minimum *P*-value approach. This database enables systematic meta-analysis of the prognostic value of a gene in multiple datasets and consequently will accelerate cancer research.

## Construction and content

### Data collection

Cancer microarray datasets with clinical annotation were intensively collected from the public domain including Gene Expression Omnibus (GEO) [8], ArrayExpress [9] and individual laboratory web sites, under the following criteria: 1) includes patient information on survival event and time, 2) contains large enough sample sizes to enable survival analysis, 3) is derived from a 'whole genome' platform and has no values missing so quantile normalization will function properly and 4) is derived from a platform for which probe annotation for a public identifier (e.g. gene symbol, GenBank accession number, UniGene ID) is available. As of February 2009, the collection included more than 40 datasets of various cancer types spanning a wide range of cancers including bladder, blood, breast, brain, esophagus, head and neck, kidney, lung, and ovarian (Table 1) [10-35], far more comprehensive than both ITTACA, which focuses on bladder cancer, breast cancer and uveal melanoma, and REMBRANDT, which specializes in brain cancers. Because some samples were used more than once by more than one study, the origin of the samples was checked. Sample duplications within a dataset were dealt with by leaving one representative arbitrary. Sample overlaps among datasets were accepted, because the study design designated by each contributor may be of value. The collected microarray datasets were standardized by using quantile normalization. Probe annotations were retrieved from GEO and ArrayExpress. Each probe was mapped to an Entrez Gene ID by querying the accompanied public identifier in UniGene database. The information in the dataset was manually curated and includes 1) study design-cohort, cancer type, subtype, endpoint, therapy history and pathological parameters-and 2) experimental procedure-sample preparation, storage, array type and signal processing method. To assess prognostic value of genes in various contexts, available endpoints such as overall survival (OS), recurrence free survival (RFS), event free survival (EFS), and distant-metastasis free survival (DMFS) were adopted as much as possible. All tables were relationally linked and stored in the MySQL server.

**Table 1:** Dataset content from PrognoScan

**Dataset**	**Cancer type**	**Subtype**	**Cohort**	**Author/Contributor**	**Array type**	**n**	**Data source**
GSE13507	Bladder cancer	Transitional cell carcinoma	Cheongju	Kim	Human-6 v2	n = 165	GEO
GSE5287	Bladder cancer		Aarhus (1995–2004)	Als *et al*. [10]	HG-U133A	n = 30	GEO
GSE12417-GPL570	Blood cancer	AML	AMLCG (2004)	Metzeler *et al*. [11]	HG-U133_Plus_2	n = 79	GEO
GSE12417-GPL96	Blood cancer	AML	AMLCG (1999–2003)	Metzeler *et al*. [11]	HG-U133A	n = 163	GEO
GSE12417-GPL97	Blood cancer	AML	AMLCG (1999–2003)	Metzeler *et al*. [11]	HG-U133B	n = 163	GEO
GSE8970	Blood cancer	AML	San Diego	Raponi *et al*. [12]	HG-U133A	n = 34	GEO
GSE4475	Blood cancer	B-cell lymphoma	Berlin (2003–2005)	Hummel *et al*. [13]	HG-U133A	n = 158	GEO
E-TABM-346	Blood cancer	DLBCL	GELA (1998–2000)	Jais *et al*. [14]	HG-U133A	n = 53	ArrayExpress
GSE2658	Blood cancer	Multiple myeloma	Arkansas	Zhan *et al*. [15]	HG-U133_Plus_2	n = 559	GEO
E-TABM-158	Breast cancer		UCSF, CPMC (1989–1997)	Chin *et al*. [16]	HG-U133A	n = 129	ArrayExpress
GSE11121	Breast cancer		Mainz (1988–1998)	Schmidt *et al*. [17]	HG-U133A	n = 200	GEO
GSE1378	Breast cancer		MGH (1987–2000)	Ma *et al*. [18]	Arcturus 22 k	n = 60	GEO
GSE1379	Breast cancer		MGH (1987–2000)	Ma *et al*. [18]	Arcturus 22 k	n = 60	GEO
GSE1456-GPL96	Breast cancer		Stockholm (1994–1996)	Pawitan *et al*. [19]	HG-U133A	n = 159	GEO
GSE1456-GPL97	Breast cancer		Stockholm (1994–1996)	Pawitan *et al*. [19]	HG-U133B	n = 159	GEO
GSE2034	Breast cancer		Rotterdam (1980–1995)	Wang *et al*. [20]	HG-U133A	n = 286	GEO
GSE2990	Breast cancer		Uppsala, Oxford	Sotiriou *et al*. [21]	HG-U133A	n = 187	GEO
GSE3143	Breast cancer		Duke	Bild *et al*. [22]	HG-U95A	n = 158	GEO
GSE3494-GPL96	Breast cancer		Uppsala (1987–1989)	Miller *et al*. [23]	HG-U133A	n = 236	GEO
GSE3494-GPL97	Breast cancer		Uppsala (1987–1989)	Miller *et al*. [23]	HG-U133B	n = 236	GEO
GSE4922-GPL96	Breast cancer		Uppsala (1987–1989)	Ivshina *et al*. [24]	HG-U133A	n = 249	GEO
GSE4922-GPL97	Breast cancer		Uppsala (1987–1989)	Ivshina *et al*. [24]	HG-U133B	n = 249	GEO
GSE6532-GPL570	Breast cancer		GUYT	Loi *et al*. [25]	HG-U133_Plus_2	n = 87	GEO
GSE7378	Breast cancer		UCSF	Zhou *et al*. [26]	U133AAofAv2	n = 54	GEO
GSE7390	Breast cancer		Uppsala, Oxford, Stockholm, IGR, GUYT, CRH (1980–1998)	Desmedt *et al*. [27]	HG-U133A	n = 198	GEO
GSE7849	Breast cancer		Duke (1990–2001)	Anders *et al*. [28]	HG-U95A	n = 76	GEO
GSE9195	Breast cancer		GUYT2	Loi *et al*. [25]	HG-U133_Plus_2	n = 77	GEO
GSE9893	Breast cancer		Montpellier, Bordeaux, Turin (1989–2001)	Chanrion *et al*. [29]	MLRG Human 21 K V12.0	n = 155	GEO
GSE11595	Esophagus cancer	Adenocarcinoma	Sutton	Giddings	CRUKDMF_22 K_v1.0.0	n = 34	GEO
GSE7696	Glioma	Glioblastoma	Lausanne	Murat *et al*. [30]	HG-U133_Plus_2	n = 70	GEO
GSE4271-GPL96	Glioma		MDA	Phillips *et al*. [31]	HG-U133A	n = 77	GEO
GSE4271-GPL97	Glioma		MDA	Phillips *et al*. [31]	HG-U133B	n = 77	GEO
GSE2837	Head and neck cancer	Squamous cell carcinoma	VUMC, VAMC, UTMDACC (1992–2005)	Chung *et al*. [32]	U133_X3P	n = 28	GEO
HARVARD-LC	Lung cancer	Adenocarcinoma	Harvard	Beer *et al*. [33]	HG-U95A	n = 84	Author's web site
MICHIGAN-LC	Lung cancer	Adenocarcinoma	Michigan (1994–2000)	Beer *et al*. [33]	HuGeneFL	n = 86	Author's web site
GSE11117	Lung cancer	NSCLC	Basel	Baty	Novachip human 34.5 k	n = 41	GEO
GSE3141	Lung cancer	NSCLC	Duke	Bild *et al*. [22]	HG-U133_Plus_2	n = 111	GEO
GSE4716-GPL3694	Lung cancer	NSCLC	Nagoya (1995–1996)	Tomida *et al*. [34]	GF200	n = 50	GEO
GSE4716-GPL3696	Lung cancer	NSCLC	Nagoya (1995–1996)	Tomida *et al*. [34]	GF201	n = 50	GEO
GSE8894	Lung cancer	NSCLC	Seoul	Son	HG-U133_Plus_2	n = 138	GEO
GSE4573	Lung cancer	Squamous cell carcinoma	Michigan (1991–2002)	Raponi *et al*. [35]	HG-U133A	n = 129	GEO
DUKE-OC	Ovarian cancer		Duke	Bild *et al*. [22]	HG-U133A	n = 134	Author's web site
GSE8841	Ovarian cancer		Milan	Mariani	G4100A	n = 83	GEO
E-DKFZ-1	Renal cell carcinoma		RZPD	Sueltmann	A-RZPD-20	n = 74	ArrayExpress

Abbreviations: AML, Acute myelocytic leukemia; DLBCL, Diffuse large B-cell lymphoma; NSCLC, Non-small cell lung cancer

### Data analysis

Survival analysis in PrognoScan employs the minimum *P*-value approach [2] to find the cutpoint in continuous gene expression measurement for grouping patients. First, patients are ordered by expression value of a given gene. Next, patients are divided into two (high and low) expression groups at all potential cutpoint, and the risk differences of the two groups are estimated by log-rank test. Then, optimal cutpoint that gives the most pronounced *P*-value (*P*_min_) is selected.

This exploratory approach, however, is known to cause inflation of a type I error because it conducts multiple correlated testing [36-38]. Thus, *P*-value correction is conducted to control the error rate using the following formula [39].



where *z *is the (1 - *P*_min _/2)-quantile of the standard normal distribution, *φ *denotes the standard normal density function, and [*ε*, 1 - *ε*] denote the range of the quantile considered to be cutpoints. PrognoScan uses *ε *= 0.1 to avoid small groupings from cutpoints of < 0.1 or > 0.9 quantile. For any given gene, this cutpoint determination and prognostic value assessment can be applied to all possible combinations of dataset, endpoint and probe. For convenience, we term each combination as "test". Note that, because probe design for each gene differs, the number of possible tests varies according to the gene. For statistical analysis and visualization, R packages  are used.

## Utility

**Figure 1:**
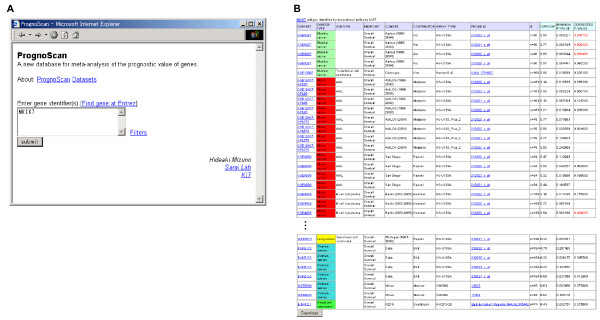
**PrognoScan screenshot and sample search results (part 1)**. (A) The top page is quite simple and only requires entering the gene identifier(s). (B) Summary table for MKI67, shown here in part (See Additional file 1 for the full table.). Column headings include dataset, cancer type, subtype, endpoint, cohort, contributor, array type, probe ID, number of patients, optimal cutpoint, *P*_min _and *P*_cor_. A statistically significant value of *P*_cor _is given in red font. Each dataset has a link to the public domain where the raw data is archived. By clicking a probe ID in the summary table, a detailed report for the test is displayed. The table can be downloaded in a tab delimited file from the button at bottom.

**Figure 2:**
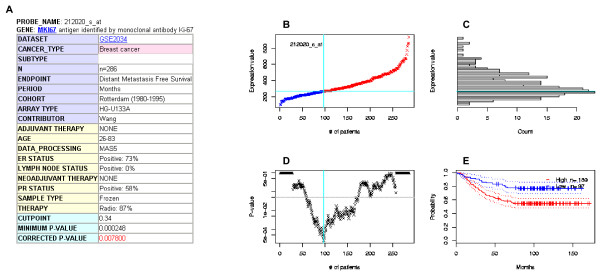
**PrognoScan screenshot and sample search results (part 2)**. (A) Annotation table. Row headings are color-coded. For example, headings of details such as therapy history, sample type and pathological parameters are highlighted in yellow and basic attributes in blue. (B) Expression plot. Patients are ordered by the expression values of the given gene. The X-axis represents the accumulative number of patients and the Y-axis represents the expression value. Straight lines (cyan) show the optimal cutpoints that dichotomize patients into high (red) and low (blue) expression groups. (C) Expression histogram. The distribution of the expression value is presented where the X-axis represents the number of patients and the Y-axis represents the expression value on the same scale as the expression plot. The line of the optimal cutpoint is also shown (cyan). (D) *P*-value plot. For each potential cutpoint of expression measurement, patients are dichotomized and survival difference between high and low expression groups is calculated by log-rank test. The X-axis represents the accumulative number of patients on the same scale as the expression plot and the Y-axis represents raw *P*-values on a log scale. The cutpoint to minimize the *P*-value is determined and indicated by the cyan line. The gray line indicates the 5% significance level. (E) Kaplan-Meier plot. Survival curves for high (red) and low (blue) expression groups dichotomized at the optimal cutpoint are plotted. The X-axis represents time and the Y-axis represents survival rate. 95% confidence intervals for each group are also indicated by dotted lines.

The top page of PrognoScan is quite simple and the user need only input gene identifier(s) (Fig. 1A). To show the features of the database and its utility, we give three meta-analysis examples. The first example is MKI67, a well known tumor proliferation marker. The prognostic value of MKI67 protein expression has been reported for many types of malignant tumor including brain, breast, and lung cancer and a few exceptions for certain tumors such as non-Hodgkin's lymphoma [40]. When given the gene, PrognoScan displays a summary in table format of tests for the gene with columns for dataset, cancer type, subtype, endpoint, cohort, contributor, array type, probe ID, number of patient, optimal cutpoint, *P*_min _and *P*_cor _as Fig. 1B for MKI67 (shown in full in Additional file 1). In the table, 52 out of 152 tests showed an association between microarray expression and cancer prognosis (bladder 3/5, blood 6/28, breast 39/83, brain 3/8, esophagus 0/1, head and neck 0/4, kidney 0/1, lung 1/16, ovarian 0/6) with 5% significance level. Clicking the probe ID in the list reveals a detailed report, which includes further annotations for the dataset (Fig. 2A) and four intuitive visualization panels (Fig. 2B–E). The example of the Rotterdam cohort for DMFS depicts that patients can be dichotomized at the 34 percentile to give the minimum *P*-value and the group with high MKI67 expression has poorer survival (*P*_cor _= 0.0078). We found all tests but one for B-cell lymphoma OS showed a positive correlation to poorer survival, consistent with previous study results [40]. We further confirmed that the expressions of other well known proliferation markers TOP2A, PCNA and Aurora A also showed association with poorer survival in various tests (Additional file 2).

**Figure 3:**
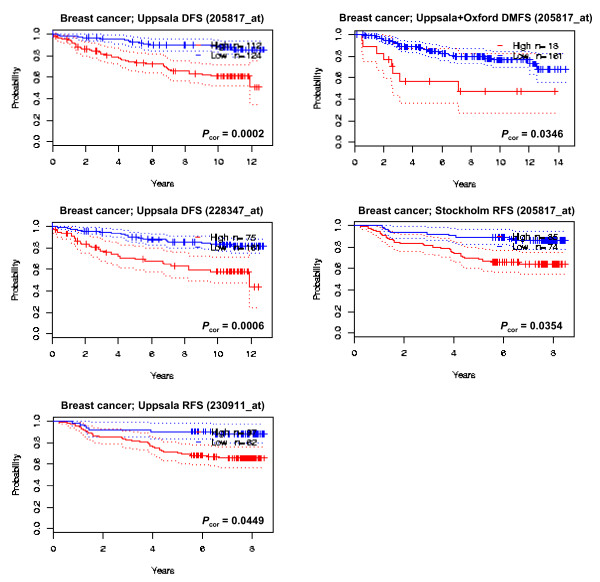
**Kaplan-Meier plots for high and low SIX1-expressing groups in breast cancers**.

The second example is SIX1, emerging as a tumor-susceptible gene. This homeobox gene has been shown to promote tumor progression through direct activation of Cyclin A1 [41,42] and to associate with prognosis of late-stage ovarian cancer [43] and hepatocellular carcinoma [44]. It has also been reported that SIX1 can be amplified and/or overexpressed in breast cancers [45,46]. Nonetheless, to our knowledge, association with breast cancer prognosis has not yet been demonstrated. And so we tested SIX1. For ovarian cancer, a clear association was not observed in three tests available in PrognoScan. For this cancer type, further subgrouping based on stage may be needed, as reported [43]. On the other hand, SIX1 expression was positively associated with 5 out of 28 breast cancer tests (Fig. 3; Uppsala cohort; *P*_cor _= 0.0002, 0.0006 and 0.0449, Uppsala+Oxford cohort; *P*_cor _= 0.0346, Stockholm cohort; *P*_cor _= 0.0354) with statistical significance, indicative of its contribution to breast cancer malignancy. In addition, SIX1 expression showed nonsignificant trend toward worse prognosis in the GUYT2 and MGH cohorts (*P*_cor _= 0.0601, 0.0729, respectively). Using PrognoScan, SIX1 expression was correlated to breast cancer prognosis in multiple tests for the first time.

**Figure 4:**
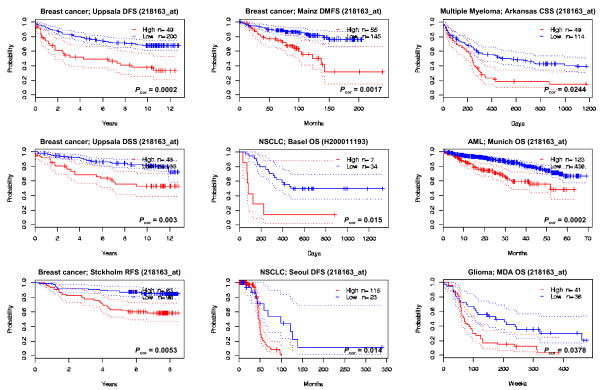
**Kaplan-Meier plots for high and low MCTS1-expressing groups in breast, lung, blood and brain cancers**.

The third example is MCTS1, a candidate oncogene amplified in T cell lymphoma. MCTS1 in a xenograft model causes transformation of NIH 3T3 mouse fibroblasts [47] and increases tumorgenicity by promoting angiogenesis and inhibiting apoptosis [48]. Similar to SIX1, prognostic analysis of this gene has not been reported for any cancers. PrognoScan depicted statistical significance in several tests: blood 2/7, breast 4/21, brain 1/2, lung 2/5 (Fig. 4). In all these 9 tests, a higher expression of MCTS1 associated with poorer survival, suggesting proactive involvement of this gene in the malignancy in the cancers. Again, this prognostic analysis was the first to show these relationships.

## Discussion and conclusion

PrognoScan is a database that focuses on the prognostic value of individual genes and differs conceptually from gene signatures. van't Veer *et al*. showed that the '70 gene signature' can predict risk of breast cancer recurrence, and that pattern analysis of multifactorial gene signature has greater potential for improving cancer subtype classification and risk prediction [49]. On the other hand, the prognostic value of an individual gene, for which pattern analysis is not applicable, suggests underlying relevance of the gene to cancer etiology and in turn stimulates research. With the number of public cancer microarray datasets with clinical annotation currently available, it is reasonable to utilize those assets to link gene expression to prognosis. Actually, Mehra *et al*., Paulson *et al*., and Kim *et al*. interrogated published cancer microarray datasets to evaluate targeted genes, GATA3, HBP1 and CUL7, respectively [50-52]. In this study, candidate oncogene SIX1 was correlated to breast cancer prognosis and MCTS1 to brain, blood, breast and lung cancer prognosis for the first time. PrognoScan aims to fulfill such substantial practical requirements.

Regarding survival analysis using publicly available microarray datasets, several considerations exist:

1) Cohorts. Datasets come from a number of different institutions around the world, and patient backgrounds differ. In addition, several datasets are based on specific subpopulations, for example, dataset GSE2034 is from lymph node-negative breast cancers, and GSE5287 is from cisplatin-containing chemotherapy-treated bladder cancers. Hence, it is possible that the specific association between gene expression and prognosis is found in a certain cohort. To give an example, Dai *et al*. reported that cell cycle genes are highly prognostic in groups with high ER expression for their age but less or nonprognostic in other groups [53].

2) Quality of care. It has been reported that the hospital itself could be a factor in clinical outcome [54-56]. This means, even if cohorts were equivalent at the time of profiling, subsequent care may affect the clinical course of a patient.

3) Experimental factors. Expression measurement of microarray is subject to various factors at the experiment level. Microdissection (e.g. GSE1378) would reduce contamination of mRNAs from non-cancer cells [57]. Formalin fixation of a sample (e.g. GSE2873) influences the quality of mRNAs [58]. Array type (e.g. Affymetrix, cDNA microarrays) and data processing method (e.g. MAS, RMA) can also influence gene expression measurements [59]. In addition, it is known that a substantial number of incorrect probes are used in microarrays [60].

4) Random error. Even though there may be no relation between a gene expression and prognosis, false positives may be detected by chance.

Thus, one needs to regard the results from PrognoScan in the context of complex conditions. Currently, PrognoScan provides curated information such as cohort, therapy history, pathological parameters and array type to aid in the interpretation of the results. As a next step, developing an "interpreter" for complex meta-analysis result is tempting and we are now contemplating the challenge. In the meantime, we will continue collecting published datasets and will update PrognoScan every 6 months. Increased data content will help the judgment of the robustness of the prognostic value of a gene.

Further plans for PrognoScan also include development of the algorithm for finding multiple cutpoints. From the limited computational resources, cutpoint selection is currently done for two-way (high and low) expression grouping. For clinical practice, three-way (high, intermediate, and low) expression grouping can also be used. Thus, we are trying to develop a grid search algorithm, demonstrated as the "X-Tile" tool [61]. In summary, this new database provides a powerful platform for evaluating potential tumor markers and therapeutic targets, and as a result, will accelerate cancer research.

## Availability and requirements

PrognoScan requires nothing other than a web browser and is available from the server at Kyushu Institute of Technology (KIT): .

## Competing interests

The authors declare that they have no competing interests.

## Authors' contributions

HM and AS designed the database. KK and KN aided in the conception and design of the database. HM and KK participated in writing the manuscript. All authors read and approved the final manuscript.

## Pre-publication history

The pre-publication history for this paper can be accessed here:



## Supplementary Material

Additional file 1 **Full summary table for MKI67**. A well known tumor proliferation marker MKI67 was assessed with PrognoScan and the summary table was indicated. Click here for file

Additional file 2 **Number of statistically significant tests for four proliferation markers among nine cancer types**. Tumor proliferation markers, TOP2A, PCNA and Aurora A were assessed with PrognoScan. Together with the result for MKI67, associations with nine cancer types were indicated. Click here for file
